# Abdominal compartment syndrome post-late Bochdalek hernia repair: A case report

**DOI:** 10.1186/1757-1626-1-199

**Published:** 2008-09-30

**Authors:** Hountis Panagiotis, Dedeilias Panagiotis, Antonopoulos Nikolaos, Bellenis Ion

**Affiliations:** 1 Department of Thoracic and Vascular surgery, Evaggelismos General Hospital, Ipsilantou 45-47, Athens, Greece; 2 Department of Cardiac surgery, Evaggelismos General Hospital, Ipsilantou 45-47, Athens, Greece

## Abstract

The aim of this case report is to discuss the rare postoperative complication of abdominal compartment syndrome in a 19-year-old Caucasian Greek male that was electively operated on for a congenital diaphragmatic hernia. The hernia was completely asymptomatic and was found in chest radiography for employment reasons. Abdominal compartment syndrome is related in most reports with trauma and abdominal operations. Timely diagnosis is key to the prevention of further organ damage and multisystem organ dysfunction because the syndrome once instituted is highly fatal.

## Background

Bochdalek diaphragmatic hernia was described in 1848, by Bochdalek [1]. It results from incomplete closure of the normal pleuroperitoneal canal during fetal development. Most cases are recognized in infancy, in utero or in childhood with respiratory symptoms. It is classically presented on the left side in 70–90% of the cases containing fat, omentum, the left kidney and various portions of the small or the large intestine. Common symptoms are pain, and occasionally nausea and vomiting from stomach or bowel obstruction. A Bochdalek hernia should always be repaired due to the high possibility of strangulation and ischemic obstruction of the herniated organs [1].

## Case report

**Figure 1:**
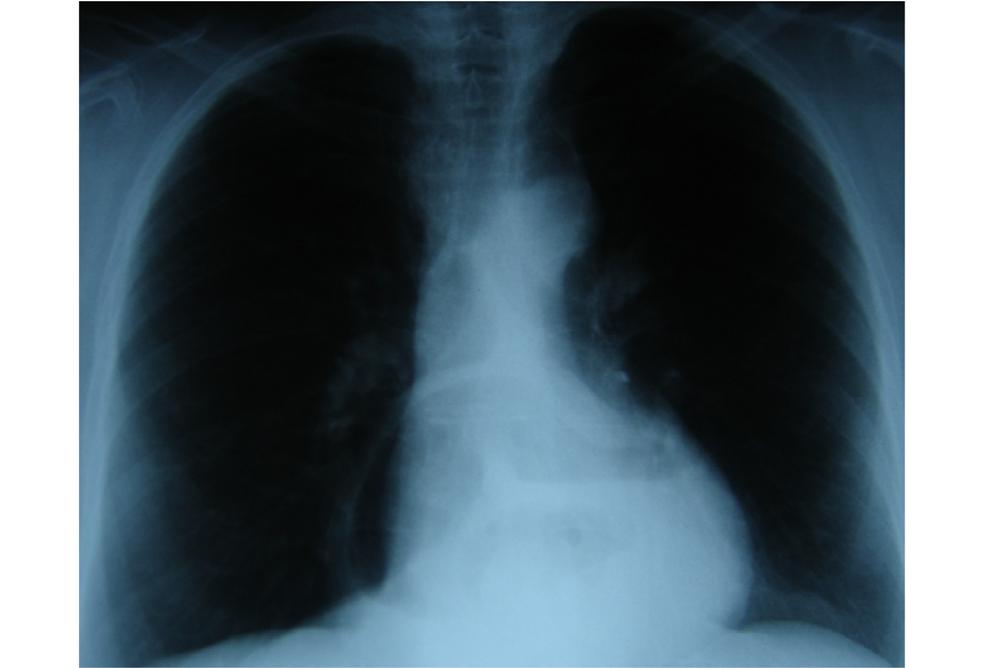
Chest x-ray showing air bubbles in the left chest.

**Figure 2:**
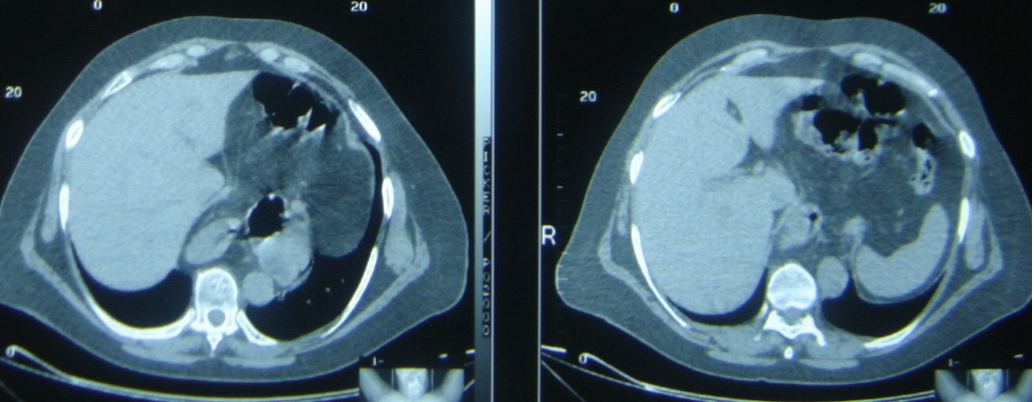
Thoracic CT scan confirmed the diagnosis of a left Bochdalek hernia.

A 19-year-old Caucasian Greek male was operated for an asymptomatic left Bochdalek hernia. The patient was a student without a history of smoke, alcohol, or medications. He was a 187 cm height and 72-kgr weight male without significant medical and family history. Chest radiography (Figure 1) that was performed for his pre-employment check up showed the possible intrathoracic presence of abdominal content and the patient was referred for Thoracic CT scan that confirmed the diagnosis of Bochdalek hernia (Figure 2).

We performed a left thoracotomy and the thoracic cavity was entered in the bed of the 5^th ^rib. The abdominal content was easily reduced from the chest back in the abdominal cavity and the diaphragm was closed with silk interrupted sutures. The patient was easily weaned and transferred to the ward. Five hours later the patient was symptomatic for abdominal heaviness, dyspnoea, chest pain and vomiting. His heart pulse was 155/min and arterial blood pressure 75/45 mm Hg. The patient was in profound respiratory distress. We transferred the patient to the operating room again and a midline abdominal incision was done. The abdominal route was preferred due to awareness of the syndrome. Small bowel loops were heavily ischemic. The abdomen remained open with proper dressing for 2 days. The patient remained in the ICU for six months. Nine months later in good condition he was discharged from the hospital. During this period he was subjected three times in partial enterectomies, four times in plastic reconstructions of the abdominal wall, and a cholocystectomy. The patient received a total of 196 units of blood during these months. Two years later the patient is in excellent condition. His case is a classic example of devastating complications even after simple thoracic operations.

## Discussion

Abdominal compartment syndrome (ACS) is an increasingly recognized clinical entity. It was first described more than a century ago and received wider recognition with the increasing repair of gastroschisis and omphalocele. These conditions, such as Bochdalek hernia and longstanding ventral hernia, are associated with insufficient room in the abdominal cavity to accommodate all of the organs without elevation of intraabdominal pressure. The syndrome is defined by an intra-abdominal pressure of greater than 25 mm Hg (or 30 cm H_2_0) with signs of end-organ compromise, confirmed by alleviation of symptoms on abdominal decompression [3]. As pressures exceed 25 mm Hg, with noted organ dysfunction, surgical decompression is indicated urgently. ACS, a late manifestation of uncontrolled IAH, causes pressure-related organ failure. Presenting signs of ACS include a firm tense abdomen, increased peak inspiratory pressures, and oliguria, all of which improve after abdominal decompression. Patients at risk for ACS include any situation with the potential to restrict the size of the abdominal compartment, including, but not limited to trauma (blunt or open); massive fluid resuscitation that results in bowel edema and distension; and the use of intraoperative packing for bleeding control, retroperitoneal hemorrhage, pancreatitis, pneumoperitoneum, and neoplasm. Although the actual incidence and mortality rate of ACS is not widely referenced the overall mortality rate with ACS is greater than 60% [4,5].

In our case we believe that pressure probably lead to inferior vena cava compression, which in turn results in decreased cardiac preload and the patient sustained a shock due to this. His course was complicated due to the intestinal ischemia and his life was in great danger after a seemingly simple repair. This syndrome should be considered in all the cases with reduction of abdominal content back in their normal position as in hernia repairs. When it is suspected intraabdominal pressure should be measured and continuously monitored. In our case although we didn't have the time to measure the abdominal pressure due to the rapid deterioration of the clinical picture of the patient. In this patient, abdominal compartment syndrome was not recognized intraoperatively or early postoperatively. The syndrome was possibly instituted gradually so he recovered well for some hours. This was probably facilitated by his age and his general condition.

## Conclusion

Early recognition of the syndrome by surgeons is the key to the prevention of further organ damage, multisystem organ dysfunction and towards reducing mortality from this highly fatal and commonly misdiagnosed syndrome.

## Abbreviations list

ACS: Abdominal Compartment Syndrome; IAH: Intrabdominal Hypertension; X-ray: classic Radiography; ICU: Intensive care unit; CT: Computed Tomography.

## Competing interests

The authors declare that they have no competing interests.

## Authors' contributions

PH was a major contributor in writing the manuscript. PD and NA analyzed and interpreted the patient data. IB took care and operated the patient. All authors have read and approved the final manuscript.

## Consent

Written informed consent was obtained from the patients for publication of this case report. A copy of the written consent is available for review by the Editor-in-Chief of this journal.
